# Practicable performance-based outcome measures of trunk muscle strength and their measurement properties: A systematic review and narrative synthesis

**DOI:** 10.1371/journal.pone.0270101

**Published:** 2022-06-17

**Authors:** Shouq Althobaiti, Alison Rushton, Ahmad Aldahas, Deborah Falla, Nicola R. Heneghan

**Affiliations:** 1 Centre of Precision Rehabilitation for Spinal Pain (CPR Spine), School of Sport, Exercise and Rehabilitation Sciences, College of Life and Environmental Sciences, University of Birmingham, Birmingham, United Kingdom; 2 Physical Therapy Department, College of Applied Medical Science, Taif University, Taif, Saudi Arabia; 3 School of Physical Therapy, Western University, London, Ontario, Canada; Mugla Sitki Kocman Universitesi, TURKEY

## Abstract

**Introduction:**

The evaluation of muscle strength is frequently used as part of the physical examination process, with decreased trunk muscle strength reported in individuals with spinal disorders (e.g., low back pain). Access to practicable performance-based outcome measures (PBOM) to monitor patients’ progress in spinal rehabilitation is essential. Knowledge of the psychometric properties of the available practicable PBOM for trunk strength evaluation is therefore needed to inform practitioners and further research.

**Objective:**

To synthesise evidence on the measurement properties of practicable measures of trunk muscle strength in adults with and without musculoskeletal pain.

**Methods:**

Following a published and registered protocol [PROSPERO CRD42020167464], databases were searched from the database inception date up to 30^th^ of June 2021. Citations and grey literature were also searched. Eligibility criteria comprised: 1) studies which examined the psychometric properties of the trunk strength outcome measures, 2) included adults ≥ 18 years, either asymptomatic or with spinal musculoskeletal pain. Non-English language studies were excluded. Two independent reviewers evaluated the quality and synthesized the data from included studies according to the COnsensus-based Standards for the selection of health Measurement Instruments (COSMIN) checklist. The overall quality of evidence was evaluated using a modified Grading of Recommendations Assessment Development and Evaluation (GRADE).

**Results:**

From 34 included studies, 15 different PBOMs were identified that have been investigated for reliability and validity, none evaluated responsiveness. In asymptomatic individuals, high quality evidence supports intra-rater reliability of digital-loading cells and moderate quality evidence supports the criterion validity of the hand-held dynamometer. Very low quality evidence exists for the reliability and validity estimates of testing tools among individuals with spinal pain.

**Conclusions:**

Findings underpin a cautious recommendation for the use of practicable PROMs to evaluate muscle strength in individuals with spinal pain in clinical practice due to the level of evidence and the heterogeneity of the protocols used. Further high quality research to explore the psychometric properties of the practicable PBOMs with detailed methodology is now needed.

## Introduction

Spinal musculoskeletal conditions are common, accounting for 21% of all causes of global disability [1]. Low back pain (LBP) and neck pain remain the two most significant causes of musculoskeletal health burden and the leading cause of long-term disability globally [2]. The management of spinal musculoskeletal conditions places an enormous economic burden on health care services worldwide [3]. In the United Kingdom for instance, LBP costs the National Health Service (NHS) around £1 billion per year [4].

The stability and mobility of the spine and extremities during functional activities depends on the activity of the trunk muscles [5, 6]. Studies have reported a link between trunk muscle weakness and spinal disorders [7–10], excessive spinal curves [11] and lower limb injuries [12]. The National Institute for Health and Care Excellence (NICE) guidelines (2016) for LBP recommend exercise programs including strengthening exercises as a clinical and cost-effective management approach [13]. Evidence supports that individual with spinal musculoskeletal conditions may benefit from strengthening exercises directed towards the trunk muscles [14, 15]. Therefore, it is essential to identify practicable Performance-based outcome measures (PBOM) of trunk muscle strength to evaluate and inform patient progression [16] and document the efficacy of rehabilitation programmes [17].

Trunk muscle strength testing is an essential part of a patient examination process, with several PBOM being used in clinical practice and research, including isokinetic dynamometers (ID), iso-station dynamometers [18], hand-held dynamometers (HHD) and manual muscle testing [19]. PBOM need to exhibit a sufficient psychometric property to accurately reflect a patient’s status and guide clinical decision making [20]. Several reviews have evaluated different PBOM of trunk muscle strength, their usefulness in routine clinical practice and to determine the hierarchy of strength values of trunk movements, from strongest to weakest, yet with little consideration of their psychometric properties [18, 21, 22]. The isokinetic and iso-station dynamometers are considered the gold standard for trunk muscle strength testing with the psychometric properties of the ID having been reviewed extensively in the literature [7, 23, 24] and acceptable levels of reliability (ICC > 0.70) and validity established. However, the ID is expensive costing around $40,000 [25], and testing multiple joints is time-consuming [26]. Moreover, with the limited portability makes the ID impractical for routine clinical testing [27]. More practical and inexpensive tools are therefore needed [3]. Thus far no evidence has summarised the psychometric properties of practicable PBOM of trunk muscle strength. The aim of this review is to evaluate the psychometric properties of practicable PBOM of trunk muscle strength.

## Methods

### Protocol and registration

A systematic review was designed in line with the COnsensus-based Standards for the selection of health Measurement INstruments (COSMIN) methodology for systematic reviews, and was conducted according to a registered [PROSPERO CRD42020167464] and published protocol [28]. Review reporting adheres to the Preferred Reporting Items for Systematic Reviews and Meta-Analyses checklist (PRISMA) [29] **(S1 Table)**.

### Eligibility criteria

Eligibility criteria were based on; sample, phenomenon of interest, design, evaluation, research (SPIDER) search concept tool [30] as detailed in **Box 1**.

**Box 1. Eligibility criteria**

**Inclusion criteria**

S- Sample:

Adults [aged ≥18 years] who are either athletic, healthy, or experiencing any spinal musculoskeletal conditions (MSK) were eligible. MSK conditions include any condition that affects the spinal bones, joints, muscles, and associated tissues such as ligaments and tendons according to the International Classification of Diseases [31] (e.g., neck pain, thoracic spine pain, low back pain (LBP), arthritis, osteoporosis, scoliosis. etc.).

PI- Phenomenon of Interest:

All practical PBOM of trunk muscle strength for use in a clinical or field-based setting, including manual, functional and mechanical methods.

D-Design:

Observational studies including cross-sectional study design were included.

E- Evaluation:

The psychometric properties based on the COSMIN Taxonomy of the clinical-based trunk strength outcome measures COSMIN taxonomy encompasses the definitions of the three main domains: reliability, validity and responsiveness [32] (S2 Table).

R-Research type: Quantitative

**Exclusion criteria**

Studies published in languages other than English.

### Information sources

The lead author (SA) conducted searches using subject headings and free text from relevant keywords identified during the scoping search as well as COSMIN recommended filters for retrieving studies on measurement properties. The following databases were searched from the database inception date up to 30^th^ June 2021: CINAHL and SPORTDiscuss (via) EBSCO interface, MEDLINE and EMBASE (through) Ovid interface, Web of Science and Pedro. Hand searching through checking reference lists of the included studies and grey literature searches including British National Bibliography and Open Grey were carried out.

### Search strategy

The search strategy was designed drawing on subject and methodological expertise of co-authors (NRH, AR and DF) and a specialist librarian. The full search strategy is described in the published protocol [28].

The following search terms were used to search in the MEDLINE database at title, abstract and the full text also, and then it was adapted for the other databases: “Trunk musc* strength”, “Trunk musc* power”, “Torso strength.”, (core strength or core power or core torque). Search filters designed by COSMIN such as (reliab* or unreliab* or valid* or coefficient or homogeneity or homogeneous or internal consistency). were also used when appropriate. MEDLINE full search string highlighted in **(S1 Fig)**.

### Selection process

After removing duplicates using the EndNote V. X9 (Clarivate Analytics), two independent reviewers (SA, AA) screened the titles and abstracts of all identified articles using the pre-identified eligibility criteria and categorising articles into ‘include’, ‘unsure’ (need full text) and ‘exclude’. The full text of the potentially relevant articles was retrieved and screened; articles were included if both reviewers reached a consensus on eligibility. A third reviewer was available to resolve any disagreements.

### Data collection process and data items

Both reviewers (SA, AA) independently extracted data from the included studies using a piloted standardised form. Data items extracted from individual studies were information regarding study characteristics, study setting, characteristics of the population, PBOM of trunk muscle strength, type of muscle contraction measured, measurement procedure, measurement properties; reliability (test-retest); (inter-rater); or (intra-rater), measurement error, validity including both criterion and construct validity, and the responsiveness, statistical methods used and results.

### Risk of bias assessment

As per the protocol [28], the COSMIN risk of bias (ROB) checklist for systematic reviews was implemented to evaluate the ROB of included studies [31]. Even though the checklist was originally designed for patient reported outcome measures, it has been recommended for adaptation and to evaluate the psychometric properties of other measures including PBOM [32]. Two reviewers (SA, AA) independently evaluated the ROB and rated each item as either ‘very good’, ‘adequate’, ‘doubtful’ or ‘inadequate’ quality [33]. The overall ROB of each measurement property was subsequently rated based on ‘the worst score counts principle’ [33].

### Data synthesis

A meta-analysis was not possible due to the heterogeneity across studies in population, measurement tools and methods of data analysis. Accordingly, a narrative synthesis was conducted in line with the COSMIN guidelines [32]. Following the ROB assessment, each outcome measure was then independently rated against COSMIN pre-determined criteria for good measurement properties [34]. Two reviewers independently synthesised the results per group (either asymptomatic or with spinal pain) and pooled the results for each measurement property (reliability, validity or responsiveness) per outcome measure.

The results were rated against the COSMIN updated criteria for good measurement properties as; sufficient ‘+’, insufficient ‘-’, inconsistent ‘±’ or indeterminate ‘?’ [2]. Based on COSMIN methodology, the results were considered as sufficient (+) when intraclass correlation coefficient (ICC) or where weighted Kappa for relative reliability was ≥0.70. For absolute reliability, the smallest detectable change (SDC) or limits of agreement (LoA) value was less than the minimal important change (MIC) [32]. For construct validity the results needed to align with the hypothesis defined by the review team; if no hypothesis was identified then the evidence was not graded, as per COSMIN recommendation [32]. With regard to the criterion validity, results were considered of sufficient validity if the correlation with a known gold standard was ≥ 0.70 or AUC ≥ 0.70, and the results needed to be in accordance with the hypothesis or AUC ≥ 0.70 to be considered of sufficient responsiveness. If the ICC, MIC, weighted Kappa, correlations, or hypothesis were not reported or not defined then the results were rated as indeterminate.

The overall level of evidence was then graded by each reviewer independently based on the modified Grading of Recommendations Assessment, Development, and Evaluation [GRADE] approach for systematic reviews where ratings were made as per ‘high’, ‘moderate’, ‘low’ or ‘very low’ [34, 35]. Four aspects of GRADE were taken into account which are: ROB, inconsistency, imprecision, and indirectness [34].

## Results

### Study selection

**Fig 1:**
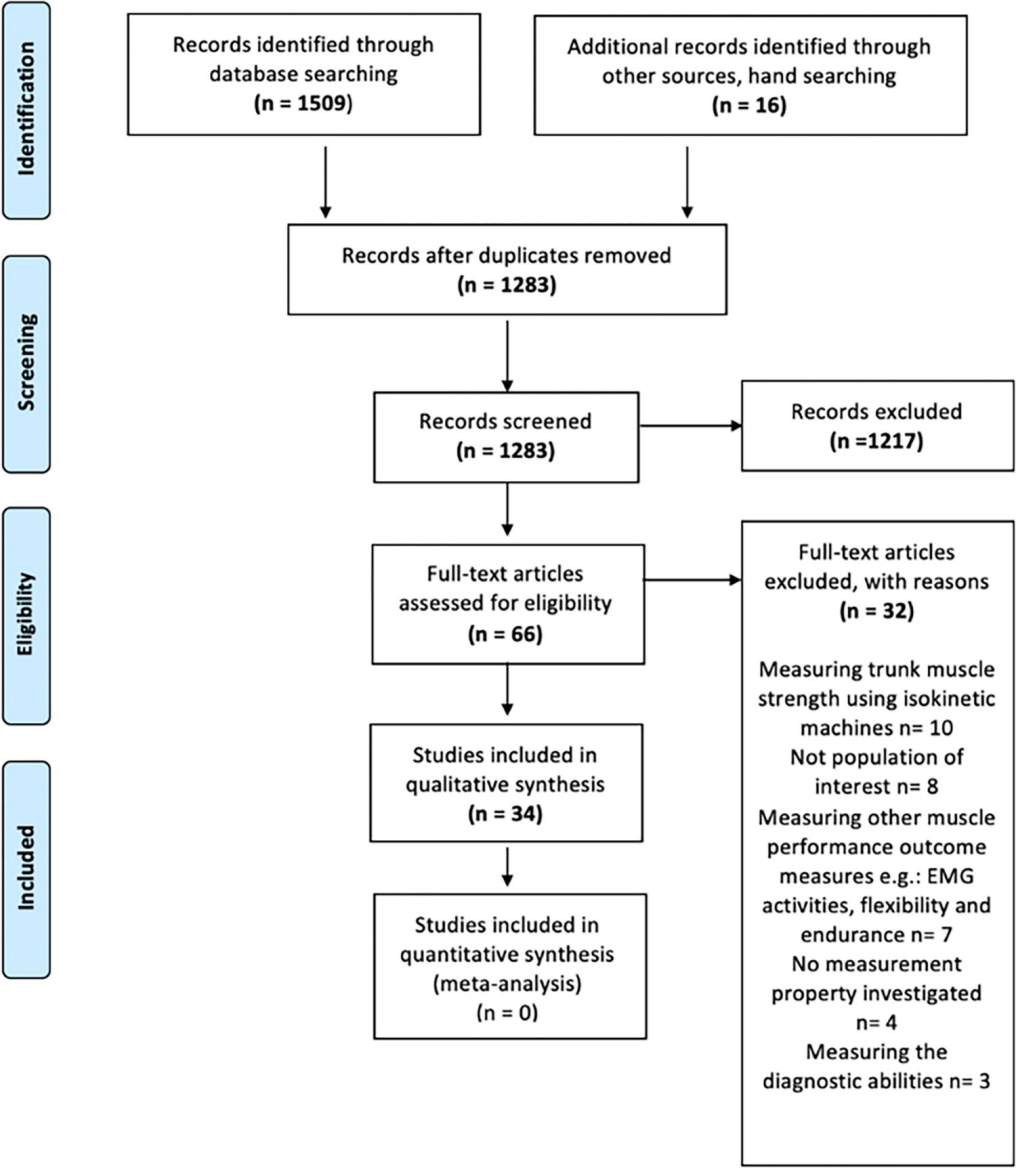
Flow diagram summarising numbers of articles included at each stage of the review.

A total of 1525 studies were identified through database and hand searching. After removing duplicates, 1283 studies were screened at the title and abstract stage. A total of 66 studies were retained for full text screening. Thirty-four studies met the eligibility criteria and were included. The detailed selection process and reasons for exclusion are highlighted in **Fig 1**.

### Study characteristics

From the 34 studies, 28 studies included asymptomatic individuals [36–63] and six investigated individuals with (LBP) [53, 54, 64–67]. One study investigated women with osteoporosis [68], and one investigated individuals with neck pain [56]. Fifteen PBOM were investigated, and to facilitate interpretation of results, they were grouped into five categories **(see Box 2)**. Reliability was evaluated in all 34 studies, criterion validity in six studies and construct validity in seven studies: with no study evaluating responsiveness. **Tables 1** and **2** summarise the characteristics of the included studies and the investigated measurement properties.

**Table 1:** Studies characteristics.

Study	Country/Settings	Sample size	Participants characteristics	Outcome measures
**Andre et al (2012)** [36]	USA	n = 23	Age: M Mean (SD): 23 (±3.16), F 20.67 (± 12.39) asymptomatic Gender: F/M: 15/ 8	Dynamometer (Fitrodyne; Fitronics, Bratislava, Slovakia)
**Azghani et al (2009)** [37]	Iran / Laboratory	n = 30	Age: Mean (SD): 25 (2.5) asymptomatic Gender: F/M: 0/30	Triaxial isometric trunk strength measurement system
**Conway et al (2016)** [64]	UK / Laboratory	Asymptomatic n = 38 CLBP n = 19	Age: Mean (SD) asymptomatic 31 (12) CLBP 28 (12) Gender: F/M: A 15 / 23, CLBP 9/ 10	Thoracic extension endurance test TEX (the Biering–Sorensen test)
**Cowley et al (2009)** [38]	USA / Laboratory	n = 8	Age: Mean (SD) F 24.4 (4)/ M 23.3 (0.58) asymptomatic Gender: F/M: 5/3	Front abdominal power test [FAPT])
**De Blaiser et al (2018)** [63]	Belgium	n = 29	Age: Mean (SD) F 21.6 (1.4), M 21.9 (1.1) asymptomatic Gender: F/M: 14/ 15	Micro FET 2 HHD
**Demoulin et al (2006)** [39]	Belgium / University hospital	n = 10	Age: Mean (SD) F 22.8 (1.3)/ M 23.6 (2.2) asymptomatic Gender: F/M: 5/ 5	David Back system
**Essendrop, Schibye, and Hansen (2001)** [40]	Denmark / Laboratory	n = 19	Age: Mean (SD): 35 (6.9) asymptomatic Gender: F/M: 13/ 6	Strain gauge dynamometers fixed to the wall
**Glenn et al. (2015)** [41]	USA/ Laboratory	Reliability n = 30 validity n = 23	Age: Mean (SD) F 23 (1.9)/ M 22.9 (1.6) asymptomatic Gender: F/M: Reliability 19/ 11 Validity 14/ 9	The abdominal test and evaluation systems tool (ABTEST)
**Graves et al (1990)** [42]	USA/ Laboratory	n = 136	Age: Mean (SD), range F 24.3 (9.1), 17–52 / M 29.4 (10.7), 18–58 asymptomatic Gender: F/M: 80/ 56	MedX lumbar extension machine
**Harding et al (2017)** [43]	Australia	n = 52	Age: Mean (SD), range 46.5 (20.5), 21–80 asymptomatic Gender: F/M: 26/ 26	HHD (Lafayette Manual Muscle Testing Systems; Lafayette, USA)
**Jubany et al (2015)** [44]	Spain/ Laboratory	n = 20	Age: Mean (SD): 27.6 (10.1) asymptomatic Gender: F/M: 11/ 9	Custom-made instrument including HHD
**Kahraman et al (2016)** [66]	Turkey/ Laboratory	n = 38	Age: Mean (SD): 35 (10) CLBP Gender: F/M: 14/ 24	HHD
**Kato et al (2020)** [45]	Japan/ Laboratory	n = 20	Age: range: (24–41 years) asymptomatic Gender: F/M: 7/ 13	Innovative exercise device
**Kienbacher et al (2014)** [46]	Austria	n = 86	Age: range **group 1** (44): 18–49, **group 2** (42): 50–90 asymptomatic Gender: F/M: 40/ 46	Davide Back system
**Kienbacher et al (2016)** [65]	Austria	n = 210	Age (no): range (median) **G1**(67): 60–90 (68.3), **G2** (81): 40–59 (49.8), G3 (62): 18–39 (27.5) LBP Gender: F/M: 112/ 98	Davide Back system
**Ladeira et al (2005)** [47]	USA	n = 28	Age: range: 21–40 asymptomatic Gender: F/M: 12/ 16	Double leg lowering manuver (DLLM)/ Nicolas HHD.
**Loss et al (2020)** [48]	Brazil	n = 15	Age: Mean (SD): 27.7 (7.1) asymptomatic Gender: F/M: 15/ 0	Portable, one-dimensional, trunk-flexor muscle strength measurement system (Measurement System)
**Mesquita et al (2019)** [49]	Brazil	n = 21	Age: Mean (SD): 64 (4) asymptomatic Gender: F/M: 21/0	Digital loading cell fixed to the wall
**Moreland et al (1997)** [50]	Canada	n = 39	Age: Mean (SD): 35 (9.3) asymptomatic Gender: F/M: 24/ 15	Micro Fit HHD (make test)
**Newman et al (2012)** [61]	Canada / research laboratory	n = 12	Age: Mean (SD): 27.9 (9.9) asymptomatic Gender: F/M: 6/6	MicroFET2 HHD
**Paalanne et al (2009)** [51]	Finland	Intra-rater reliability n = 19 Inter-rater reliability n = 15	Age: Mean (SD), range 22.7 (2.9), 19 30 Asymptomatic Gender: F/M:/?	Computerized strain gauge dynamometer
**Park et al (2017)** [52]	Korea	n = 30	Age: Mean (SD) F 33.1 (5.5)/ M 34.8 (7.5) asymptomatic Gender: F/M: 15/ 15	HHD (Power TrackII Commander Muscle Tester) Fixed on specially designed chair
**Pienaar and Barnard (2017)** [53]	South Africa	LBP n = 42 Healthy n = 24 LBP n = 18	Age: Mean (SD): 47.58 (18.58) asymptomatic LBP Gender: F/M: Healthy 12/ 12, LBP 9/ 9	A pressure air biofeedback device (PABVR)
**Pitcher, Behm, and MacKinnon (2008)** [54]	Canada	n = 20 Healthy n = 10 LBP n = 10	Age: Mean (SD): asymptomatic 24.7 (2.9) LBP 29.1 (8.2) Gender: F/M: 0/ 20	Strain gauge dynamometer
**Roussel et al (2006)** [55]	Belgium/ Ambulatory care in hospital	n = 61	Age: Mean (SD): 36.9 (13.1) asymptomatic Gender: F/M: 30/31	Tergumed equipment (4 devices for each movement)
**Roussel et al (2008)** [67]	Belgium/ Ambulatory care in hospital	n = 12	Age: Mean (SD): 40.2 (11.5) CLBP Gender: F/M: 7/5	Tergumed equipment (4 devices for each movement)
**Sasaki et al (2018)** [69]	Japan	n = 24	Age: Mean (SD): 21.1 (2.5) asymptomatic Gender: F/M: 8/ 16	Portable trunk muscle torque measurement instrument (PTMI).
**Scheuer and Friedrich (2010)** [56]	Austria	NP n = 53 Healthy n = 42	Age: Mean (SD): NP 49.7 (10.74) asymptomatic 48.7 (12.02) Gender: F/M: 73%/ 27%	Back check
**Sell et al (2015)** [57]		n = 20	Age: Mean (SD): 22.7 (4.8) asymptomatic Gender: F/M: 10/ 10	Portable trunk muscle torque measurement instrument (PTMI).
**Steeves et al (2019)** [62]	Canada/ laboratory	Reliability n = 10 Validity n = 20	Age: Mean (SD) Reliability 25 (3.8) Validity M 25.4 (5.0)/ F 23.9 (4.0) asymptomatic Gender: F/M: 14/ 16	Novel trunk maximal isometric force assessment
**Tarca et al (2020)** [58]	Australia	Reliability n = 31 Validity n = 35	Reliability: Age: Mean (SD) 35.2 (18.0) years (52% males) Validity: Age: Mean (SD) 33.9 (17.3) years, (57% males).	A Lafayette HHD
**Udermann, Mayer, and Murray (2004)** [59]	USA	n = 60	Age: Mean (SD) F 21 (2.2)/ M 22.6 (2.7) asymptomatic Gender: F/M: 30/ 30	BackUP ^TM^ lumbar extension dynamometer
**Valentin and Maribo (2014)** [68]	Denmark	n = 48	Age: Mean (SD): 72 (9.3) Osteoporosis Gender: F/M: 48/ 0	HHD (Power Track II commander).
**Yang et al (2020)** [60]	China	n = 60	Age: Mean (SD): 61.7 (7.6) asymptomatic Gender: F/M: 50/ 10	HHD microFET3, tension (externally fixed)

SD = Standard deviation, F = Female, M = Male, HHD = Hand-held dynamometer, CLBP = Chronic low back pain, NP = Neck pain,? = Unknown.

**Table 2:** Summary of measurement properties.

Study	Measurement property	No. of raters and testing schedule	Groups	Position/ fixation/ resistance	Movement measured/ Muscle contraction	Statistical measures	Results
**Andre et al (2012)** [36]	Between days test re-test reliability.	1 rater Between days Time interval: 3–7 days	Asymptomatic	Sitting / no fixation/ resistance from the weight stack from the pulley system.	Rot Isotonic-Concentric	ICC	ICC 0.93–0.97
**Azghani et al (2009)** [37]	Test re-test reliability.	1 rater Within day Time interval: 2 min	Asymptomatic	Standing fixation from thoracic pad and pelvic pad.	Flex, Ext, R & L lateral flexion R & L Rot/ Isometric	ICC _(2,1)_, SEM	ICC 0.69–0.91, SEM = 7.4–23.9
**Conway et al (2016)** [64]	Construct validity: (Convergent)	One rater Within day Time interval: 10 Sec between contractions, 72 h between sessions.	CLBP	Sitting, fixation around pelvic, thighs and feet. Resistance over the thoracic spine.	Ext/ Isometric	Pearson’s correlation	Combined groups: weak positive correlation r = 0.035, CLBP r = 0.120. A non-significant very weak negative correlation asymptomatic group r = -0.060
**Cowley et al (2009)** [38]	Test-re- test reliability.	One rater Between days Time interval: 2 min between trials/ S 1 and S 2 were separated by 7 days, whereas S 2 and 3 were separated by 2 days.	Asymptomatic	Supine with knees 90˚ flex/ curl bar secured the feet to the ground/ resistance of 2-kg medicine ball.	Flex. Isotonic—concentric.	ICC _(2,1)_ (95% CI) LOA using Bland Altman Plots.	ICC = 0.95 (0.83–0.99), LoA = session 1 vs 2 = 15% LoA = session 2vs 3 = 17%
**De Blaiser et al (2018)** [63]	Intra-rater, inter-rater reliability & criterion validity:	Two raters Between days Time interval: 2 weeks between sessions/ 1 h between testers / 15 sec between trials.	Asymptomatic	Supine for (Flex), prone for (Ext)/ fixation around lateral malleolus and ASIS, PSIS. Resistance below the suprasternal notch for Flex & at the height of T4 for Ext.	Flex (0˚ & 30˚), Ext (0˚ & 30˚)/ Isometric	ICC _(2,1)_ (95% CI), SEM, LOA, Pearson’s correlation	**Intra-rater:** Flex (0˚) ICC = 0.67 (0.4–0.83), SEM = 26.41 Flex (30˚) ICC = 0.9 (0.8–0.95), SEM = 17.69 Ext (0˚) ICC = 0.93 (0.92–0.98), SEM = 14.42 Ext (30˚) ICC = 0.8 (0.16–0.93), SEM = 33.02 **Inter-rater**: Flex (0˚) ICC = 0.78 (0.28–0.91), SEM = 17.98 Flex (30˚) ICC = 0.93 (0.82–0.97), SEM = 16.45 Ext (0˚) ICC = 0.76 (0.56–0.88), SEM = 20.88 Ext (30˚) ICC = 0.82 (0.17–0.94), SEM = 31.53 LOA = (-4.78 Nm- 18.61 Nm), r = 0.64–0.85).
**Demoulin et al (2006)** [39]	Test-re- test, inter-rater, & inter site reliability.	Two raters Between days Time interval: 7–10 days between sessions / 45 sec between trials	Asymptomatic	Sitting with fixation around pelvic, thighs and against tibial tuberosity, Additional shoulder pads were used for Flex and Rot testing. Resistance against the upper back.	Flex, Ext, Rt & L lateral flexion, R & L Rot/ Isometric.	CV %	Test re-test CV% = 3.4–7.6%, Inter-rater CV% = 3.4–8.1%, Inter site reproducibility CV% = 4.2–12.7%.
**Essendrop, Schibye, and Hansen (2001)** [40]	Test-re- test reliability.	One rater Between days Time interval: 1week between sessions and 30 sec between trials.	Asymptomatic	Standing with fixation around pelvic. Resistance around the shoulders.	Flex, Ext/ Isometric	ICC (95% CI), Pearson correlation.	Force measurements: Flex ICC = 0.97 (−42.1 to −1.5), r = 0,97 Ext ICC = 0.93 (−76.4 to −19.5), r = 0.95 Torque measurements: Flex ICC = 0.96 −6.6–8.9), r = 0,96 Ext ICC = 0.91 −28.3 to −4.4), r = 0.94
**Glenn et al. (2015)** [41]	Test re-test reliability & convergent validity.	One rater Within day Time interval: 5 min between trials.	Asymptomatic	Supine with the knees and hips at 90° Flex with no fixation. Resistance over the xiphoid process.	Flex / Isometric	ICC (95% CI), Pearson’s correlation	Max force ICC = 0.753 (0.544–0.875) Max power ICC = 0.893 (0.789–0.948). Correlation trial 1 and 2 r = 0.84 Correlation between the 1 RM with average power r = 0.70.
**Graves et al (1990)** [42]	Test re-test reliability.	One rater Within day & between days. Time interval: 72 hours between sessions and 20–30 min between trials.	Asymptomatic	Sitting (0°, 12°, 24°, 36°, 48°, 60°, 72°) of Ext. Fixation around pelvic, thighs and against tibial tuberosity. Resistance against the upper back.	Ext /Isometric	Pearson product-moment correlation, SEM	Within day (n = 136) D1 r = 0.78–0.96, D2 r = 0.94–0.98, D1 SEM = 37.6–46.9 Nm. D2 SEM = 28.7–34.4 N.m. Between days (n = 119) D1 & D2 r = 0.70–0.95, D2 & D3 r = 0.81–0.97. SEM (D1 & D2) = 40.2–54.1 N SEM (D2 & D3) = 32.8–46.3 N.
**Harding et al (2017)** [43]	Test re-test reliability & criterion validity.	One rater Within day & between days. Time interval: 30 s rest between trials / 7 days rest between sessions	Asymptomatic	Standing with fixation 1 cm below ASIS. Resistance over the Seventh thoracic vertebrae.	Ext /Isometric	ICC (95% CI), LOA, Pearson’s correlation	Trial 2 vs trial 3: S1 ICC = 0.98 (0.97–0.99), S2 ICC = 0.96 (0.94–0.98). S1 vs S2: ICC = 0.901 (0.833–0.943). LOA = −6.63 to 7.70 kg, with a mean bias of +0.71 kg. r = 0.82–0.85.
**Jubany et al (2015)** [44]	Test re-test reliability & construct validity (Convergent).	One rater Within day. Time interval: 1 min between trials.	Asymptomatic	Standing, with fixation around pelvic and resistance over thoracic spine.	Flex, Ext, side bending / Isometric	ICC (95% CI), SEM, Pearson’s correlation	ICC = 1.0 (0.9–1), SEM = 0.3–0.8, r = 0.7–0.8
**Kahraman et al (2016)** [66]	Test re-test reliability.	One rater Between days. Time interval: 30 sec between trials and 48–72 hours between sessions.	CLBP	A 30˚ reclined position for Flex, and prone position for Ext. No fixation when measuring Flex and around hips and below knees when measure the Ext. Resistance for Flex, 1 inch below the sternal notch, for Ext, at the level of the inferior angle of the scapula.	Flex, Ext/ Isometric/	ICC _(2,1)_ (95% CI) SEM, MDC, CV	Flex: ICC = 0.74 (0.56–0.86), SEM = 1.88, MDC = 5.21, CV = 26.41 Ext: ICC = 0.65 (0.42–0.80), SEM = 1.2.71, MDC = 7.51, CV = 27.62.
**Kato et al (2020)** [45]	Intra-rater & inter-rater reliability.	Time interval: Inter-rater: 1 hour between raters. Intra-rater one week.	Asymptomatic	Sitting with back straight and feet supported and arms at the sides. No fixation. Resistance from the pressure of the device.	Abdominal strength	ICC, SEM	**Intra-rater**: ICC = 0.95, 95% CI: 0.87–0.98, SEM = 2.0. **Inter-rater:** ICC = 0.99, 95% Cl: 0.96–0.99), SEM = 1.0
**Kienbacher et al (2014)** [46]	Test re-test reliability.	Between days. Time interval: 1–2 days between S1 & S2, 6 weeks between S2 & S3/ 15 Sec between trials.	Asymptomatic	Sitting upright to measure Flex, for Ext trunk flexed 30^o^ anteriorly, for Rot upper body upright and lower body rotated to the opposite side by 30^o.^ Fixation around back, pelvic, knees and feet, additional shoulder pads were used for Flex and Rot testing. Resistance over the upper back for Ext, over the shoulders for Flex and Rot.	Flex, Ext, R & L. Rot/ Isometric	ICC _(2,1)_, SEM/ SRD%	**Short term**: Ext ICC = 0.83–0.85, Flex ICC = 0.87–0.94, Rot ICC = 0.81–0.89 **Long term:** Ext ICC = 0.78–0.91, Flex ICC = 0.81–0.91 Rot ICC = 0.80–0.87. **Short term**: Ext SRD%/ 22.27–28.92% Flex SRD% = 15.48–25.88% Rot. SRD% = 30.61–44.70%. **Long term**: Ext SRD% = 21.2–32.68% Flex SRD% = 18.49–27.00% Rot. SRD% = 33.29–44.99%.
**Kienbacher et al (2016)** [65]	Test re-test reliability.	Between days. Time interval: 1–2 days between S1 & S2, 6 weeks between S2 & S3/ 15 Sec between trials.	LBP	Sitting upright to measure Flex, for Ext trunk flexed 30^o^ anteriorly, for Rot upper body upright and lower body rotated to the opposite side by 30^o^. Fixation around back, pelvic, knees and feet, additional shoulder pads were used for Flex and Rot testing. Resistance over the upper back for Ext, over the shoulders for Flex and Rot.	Flex, Ext, R. & L. Rot/ Isometric	ICC _(2,1)_, SEM, SRD%	**Short term**: ICC = 0.86–0.96, SEM = 8.58–21.16, SRD% = 18.60–50.57%, **Long term:** ICC = 0.84–0.96, SEM = 9.53–24.43, SRD% = 20.84%- 51.92%.
**Ladeira et al (2005)** [47]	Test re-test reliability & construct validity (Convergent).	four raters Within day Time interval: 15 min between S1 and S2/ 30 sec between trials.	Asymptomatic	DLLM: Supine with hips flexed to 90^o^, knees and ankles in neutral position. HHD: supine with hips and knees at 90^o^. No fixation, no resistance for DLLM, at the sternum during HHD measurement.	Flex / DLLM: Eccentric/ HHD: Isometric	ICC _(1,1)_, (95% CI) Pearson’s correlation	**DLLM** ICC _(1,1)_ = 0.955 (0.90–0.97), r = 0.93 **HHD** r = 0.968, Correlation r = -0.33 and -0.44.
**Loss et al (2020)** [48]	Test–retest reliability & construct validity.	Within day & between days. Time interval: 24 h between days test re-test/ 2 min between trials.	Asymptomatic	Supine with knees and hips flexed, with no fixation. Resistance at the level of axilla.	Flex / Isometric	t test (for construct validity). ICC _(1, 2)_, SEM, MDC.	p = 0.10–0.44 ICC = 0.99, SEM = 0.1–1.6, MDC = 0.3–3.1N.
**Mesquita et al (2019)** [49]	Test re-test reliability.	One rater Between days. Time interval: 15 sec rest between trials and 48 h rest between sessions.	Asymptomatic	Upright sitting with fixation around legs. Resistance for Ext over the scapula, for Flex at the level of xiphoid process.	Flex, Ext/ Isometric.	ICC _(2,1)_ SEM, MDD	ICC = 0.86–0.93, SEM = 17.6–18.4, MDD = 48.9–51.1.
**Moreland et al (1997)** [50]	Inter rater reliability.	Three raters Between days. Time interval: 30 sec between trials and 3 sessions in 3 dyes.	Asymptomatic	Flex (Semi reclined position 30˚), Ext (prone with trunk out of the table). No fixation during Flex, for Ext (around hips and below knees). Resistance applied 1 inch below the sternal notch during Flex measurement, and at the level of the inferior angle of the scapula during Ext.	Flex, Ext/ Isometric.	ICC _(2,1)_ SEM	ICC = 0.24–0.25, SEM = 60–68 N.
**Newman et al (2012)** [61]	Intra-rater & inter-rater reliability	Two raters Between days. Time interval: More than 24 h between sessions and 30 sec between trials.	Asymptomatic	Sitting with fixation around pelvic. Resistance on the lateral aspect of the trunk, at the level of the axilla or mid trunk.	Side bending (dominant)/ Isometric	ICC _(2,1)_, (95% CI), SEM	Axilla position: Intra-rater ICC = 0.5–0.7 Inter-rater ICC = 0.7–0.8 SEM = 0.22 Nm/kg. Mid trunk position: Intra-rater ICC = 0.80–0.86 Inter-rater ICC = 0.87–0.88 SEM = 0.09 Nm/kg.
**Paalanne et al (2009)** [51]	Intra-rater & inter-rater reliability.	Two raters Within & between days. Time interval: Intra-rater (7 days) between sessions, Inter-rater (30 min between sessions).	Asymptomatic	Standing with fixation against the popliteal fossa, lumbar spine, and on scapular level during Flex and Rot assessment. For Ext, the fixation against proximal part of tibia, pelvis, and sternum. Resistance during Flex & Rot was positioned against chest and over the spine of the scapula during Ext measurement.	Flex, Ext, R & L Rot/ Isometric.	ICC _(1,1)_, ICC _(3,1)_ 95% LOA.	Intra-rater: ICC _(1,1)_ = 0.84–0.95, 95% LOA = -8.37, 7.47- -18.81, 14.11, Inter-rater: ICC _(3,1)_ = 0.84–0.88, 95% LOA = -9.52,7.96- -29.40, 18.16.
**Park et al (2017)** [52]	Test re-test reliability & criterion validity.	Between days Time interval: 2 h between HHD testing and Isokinetic.	Asymptomatic	Sitting with fixation around pelvic. Resistance at T7 spinous process.	Ext/ Isometric	ICC, (95% CI) Pearson’s correlation	ICC = 0.82 (0.65–0.91) r = 0,65
**Pienaar and Barnard (2017)** [53]	Test re-test reliability & construct validity (Convergent).	One rater Between days Time interval: 1 min rest between trials	Asymptomatic & LBP.	Sitting with fixation around the thorax and resistance just below the inferior angles of the scapula.	Ext/ Isometric	ICC, SEM, Pearson’s correlation	ICC = 0.99, SEM = 1.55, PAB (mb) Day 1 EMG (lV): both groups r = .75, Asymptomatic = .75, LBP = .26 Day 2: r = Whole .63, Asymptomatic = .73, LBP = (-.11)
**Pitcher, Behm, and MacKinnon (2008)** [54]	Test re-test reliability.	One rater Between days Time interval: 24–72 h between sessions / 2 min between trials.	Asymptomatic & LBP.	Prone with fixation around the lower legs, thighs, and mid-buttocks. Resistance at the level of T6- T7.	Ext/ Isometric	ICC	Asymptomatic ICC = 0.98. LBP ICC = 0.80.
**Roussel et al (2006)** [55]	Inter-rater, intra-rater reliability.	Two raters Within day Time interval: 15 min between sessions/ 10 sec between trials	Asymptomatic	Sitting with knees flexed 20° to 40° for Flex & Ext and 90° for Rot & lateral flexion. Fixation around pelvis, the trunk, and the legs.	Flex, Ext, R & L lateral flexion, R & L Rot/ Isometric.	ICC_(2,1)_, (ANOVA)	Inter-rater ICC _(2,1)_ = 0.95–0.97, Intra-rater F = 0.03–5.04.
**Roussel et al (2008)** [67]	Inter-rater reliability.	Two raters Between days Time interval: 1–7 days between sessions.	CLBP	Sitting with knees flexed 20° to 40° for Flex & Ext and 90° for Rot & lateral flexion. Fixation around pelvis, the trunk, and the legs.	Flex, Ext, R & L lateral flexion, R & L Rot/ Isometric.	ICC_(2,1)_ SEM	ICC = 0.93–0.97 SEM = 26.7–51.7.
**Sasaki et al (2018)** [69]	Criterion Validity.	One trial on each device.	Asymptomatic	Sitting with fixation around the pelvic and proximal to the knees. **Resistance** for Flex: against sternum, For Ext: against upper back.	Flex & Ext.	ICC _(2,1)_	Flex ICC = 0.807, Ext ICC = 0.789
**Scheuer and Friedrich (2010)** [56]	Inter-rater and intra-rater reliability.	Tow raters Within day & between days Time interval: Session 1 and 2 on day 1 separated by 30–60 min, 3–5 days then another 2 sessions on day 2 separated by 30–60 min)	Asymptomatic & NP individuals	Standing with fixation around the pelvic and resistance at sternum level.	Flex, Ext, R & L Side bending / Isometric.	ICC, ANOVA	Test retest: NP ICC = 0.82–0.89 Asymptomatic ICC = 0.80–0.88, Inter-rater: NP ICC = 0.00–0.36 Asymptomatic ICC = 0.00, Short term intra-rater: NP ICC = 0.00–0.41 Asymptomatic ICC = 0.00, Long term intra-rater: NP ICC = 1.12–1.49, Asymptomatic ICC = 1.73–7.21.
**Sell et al (2015)** [57]	Test re-test reliability & criterion validity.	Between days Time interval: 2 sessions separated by 24 h.	Asymptomatic	Tall kneeling position with no fixation and resistance from the medicine ball.	Flex, Ext, R & L Rot/ Concentric.	ICC (_2,1)_, (95% CI) SEM, Pearson’s correlation	Flex ICC = 0.83 (0.60–0.93), SEM = 26.02 Ext ICC = 0.83 (0.59–0.93), SEM = 30.16 Right. Rot ICC = 0.87 (0.66–0.94), SEM = 24.5 Left, Rot ICC = 0.90 (0.7–0.9), SEM = 17.44 r = 0.047–0.180.
**Steeves et al (2019)** [62]	Test re-test reliability & Construct validity.	Between days Time interval: 24 h between sessions (day 1 and day 2)/ 4 min between movements.	Asymptomatic	Sitting with fixation around feet. Resistance inferior to axilla region during Flex, and over the shoulder during rotation movement.	Flex, Rotational push (R and L), Rotational pull (R and L), Kayak-Stroke Simulation/ Isometric.	ICC, Pearson’s correlation	Within day reliability: ICC = 0.91–0.99 Between days reliability: ICC = 0.95–1.00 r = -0.22- -0.82
**Tarca et al (2020)** [58]	Test re-test reliability & Criterion validity.	Between days Time interval: 7 days for reliability, 15 min for validity.	Asymptomatic	25° reclined supine position, 25°hip Flex with knee extended on the plinth. No fixation and resistance over the sternum just below the sternal notch.	Abdominal Flex strength	ICC (95% CI) SEM, LOA,	Reliability: ICC = .86, CI = (.72-.93) SEM = 4.7 N.m. Validity: ICC = .82 (.57–.92). (P = .001). LOA = 37.5 to −19.7 N·m.
**Udermann, Mayer, and Murray (2004)** [59]	Test re-test reliability.	Between days Time interval: 24 h between sessions and 30 sec between test angles.	Asymptomatic	Sitting with fixation around the pelvic and thighs. Resistance over the spine of the scapula.	Ext/ Isometric.	ICC	ICC = 0.92–0.97
**Valentin and Maribo (2014)** [68]	Test re-test reliability.	Between days, 2 sessions/ 2 protocols (tester and tripod). Time interval: 7 days between sessions/ 60 sec between trials / 5 min between protocols.	Osteoporosis	Prone position with no fixation.	Ext/ Isometric.	ICC _(1,2),_ 95% CI SEM,	Tester fixated: ICC = 0.75 (0.63–0.88), SEM = 27.1. Tripod fixated: ICC = 0.90 (0.84–0.95), SEM = 20.5.
**Yang et al (2020)** [60]	Test re-test reliability & criterion validity.	Between days Time interval: A week interval between sessions / 1 min interval between trials and 1 h rest between each testing positions.	Asymptomatic	From three positions: sitting, standing and prone. Fixation around the pelvic (standing), around thighs (sitting), no fixation (prone). Resistance at the level of the xiphoid process (standing)/ over the superior borders of the scapula (sitting)/ At the level of scapula (prone).	Ext/ Isometric.	ICC (95% CI) SEM, LOA, CV, Pearson’s correlation	Sitting: ICC = 0.90 (0.83–0.94) SEM = 13.2, LOA = -37.83, 44.94, CV = 36.6. Standing: ICC = 0.92 (0.87–0.95), SEM = 25.5, LOA = -70.35, 90.15, CV = 20.3. Prone: ICC = 0.93 (0.88–0.95), SEM = 13.0, LOA = -35.01, 47.10. r = 0.32–0.54

Abbreviations: ICC = Intraclass correlation coefficient, 95% CI = 95% confidence interval, SEM = Standard Error of Measurement, r = Pearson’s correlation, LoA = Limits of agreement, MIC = Minimal important change, MDD = Minimum detectable difference, SRD = Smallest real difference, CV = Coefficient of variation, SDC = Smallest detectable change. CLBP = Chronic low back pain, NP = Neck pain, Flex = Flexion, Ext = Extension, Rot = Rotation. DLLM = Double leg lowering manoeuvre, HHD = Hand-held dynamometer. MBTs = Medicine ball toss tests, h = hour, min = minute, N.m = Newton per meter, kg = kilogram, Nm/kg = Newton per kilogram, S = session, D = day.

**Box 2. Categories of the included PBOM**

***1/ Hand-held dynamometer (HHD)*.**
***2/ Digital loading cells*.**
***3/ Specialised and commercialised equipment*:**
    • ***David Back®***
    • ***MedXTM***
    • ***Tergumed®***
    • ***BackUpTM lumbar extension dynamometer***
    • ***Back Check 607***
***4/ Field tests (functional tests)*:**
    • ***Front abdominal power test (FAPT)***
    • ***Medicine ball toss tests***
    • ***Double leg lowering manoeuvre (DLLM)***
***5/ Novel devices*:**
    • ***Triaxial isometric trunk muscle strength measurement system***
    • ***Pressure air biofeedback (PAB®)***
    • ***Portable trunk muscle torque measurement instrument (PTMI)*.**
    • ***Innovative exercise device***

### Risk of bias in individual and across studies

**Table 3** illustrates the ROB for each population and outcome measures. Of those studies that measured reliability, six studies exhibited adequate ROB [40, 43, 54, 57, 62, 68], four had very good ROB for criterion validity [43, 57, 60, 63], and one rated as very good ROB for construct validity [47, 52]. The remaining studies were rated as either doubtful or with inadequate ROB [37, 39, 41, 42, 45, 47–53, 56, 58, 60, 61, 63, 65, 67].

**Table 3:** Summary of risk of bias, criteria for good measurement properties and overall quality of evidence (GRADE).

Measurement property	Study	Risk of bias (ROB)	Characteristics of good measurement property	Overall rating	GRADE (quality of evidence)
**Hand-held dynamometer (HHD)- Asymptomatic individuals**					
**Reliability** **(Intra-rater).**	Harding et al (2017) [43] (Ext)	Adequate	+	**Sufficient (+)**	**Very low-quality evidence.** (-2 Very serious ROB, there are multiple studies of inadequate quality, -1 for inconsistency.)
	Jubany et al (2015) [44] (Ext, Flex, Lat.flex)	Inadequate	+		
	Ladeira et al (2005) [47] (Flex)	Inadequate	+		
	Park et al (2017) [52] (Ext)	Inadequate	+		
	Yang et al (2020) [60] (Ext)	Inadequate	+		
	Tarca et al (2020) [58]	Inadequate	+		
	De Blaiser et al (2018) [63] (Ext & Flex (0˚, 30˚)	Inadequate	± Ext & Flex 30˚ (+) Flex 0 ˚ (-)		
	Newman et al (2012) [61] (Lat. Flex)	Inadequate	± At axilla (-) Mid trunk (+)		
Reliability (Inter rater)	Moreland et al (1997) [50] (Flex, Ext)	Inadequate	-	**(±)** **Inconsistent**	**Very low-quality evidence** (-2 Very serious ROB as there are multiple studies of inadequate quality available, inconsistency and imprecision sample size <50–100)
	De Blaiser et al (2018) [63] (Flex, Ext)	Inadequate	+		
	Newman et al (2012) [61] (Lat.flex)	Inadequate	+		
Measurement error	Harding et al (2017) [43] (Ext)	Adequate	MIC (?)	**Indeterminate (?)**	**Not graded**
	Jubany et al (2015) [44] (Ext, Flex, Lat. Flex)	Inadequate	MIC (?)		
	Moreland et al (1997) [50] (Flex, Ext)	Inadequate	MIC (?)		
	Yang et al (2020) [60] (Ext)	Inadequate	MIC (?)		
	Tarca et al (2020) [58]	Inadequate	MIC (?)		
	De Blaiser et al (2018) [63] (Flex, Ext)	Inadequate	MIC (?)		
	Newman et al (2012) [61] (Lat. Flex)	Inadequate	MIC (?)		
Construct validity (convergent validity)	Jubany et al (2015) [44] **(HHD vs. BC****)**	Inadequate	+	**Sufficient (+)**	**Very low-quality evidence** (-3 Extremely serious ROB, as there is only one study of inadequate quality available, -2 Imprecision sample size <50)
Criterion validity	Harding et al (2017) [43]. (Ext)	Very Good	+	**(+)** **Sufficient**	**Moderate- quality evidence** (-1due to serous inconsistency)
	Park et al (2017) [52] (Ext)	Inadequate	-		
	Yang et al (2020) [60] (Ext)	Very Good	-		
	Tarca et al (2020) [58] (Flex)	Inadequate	+		
	De Blaiser et al (2018) [63] (Ext & Flex)	Very Good	Flex 0˚, 30˚ (+) Ext 0˚ (-), 30˚ (+)		
**Hand-held dynamometer (HHD)- Spinal musculoskeletal pain**					
*LBP* Reliability (Intra-rater).	Kienbacher et al (2016) [65] (Ext) (Flex)	Inadequate	- +	**(±)** **Inconsistent**	**Very low-quality evidence** (-3 Extremely serious ROB, as there is only one study of inadequate quality available, -1 for inconsistency and -2 for imprecision sample size <50)
Measurement error	Kienbacher et al (2016) [65]	Inadequate	MIC (?)	**Indeterminate (?)**	**Not graded**
*Osteoporosis* (Reliability Intra-rater).	Valentin and Maribo (2014) [68] (Ext)	Adequate	+	**Sufficient (+)**	**Very low-quality evidence**. (-1 Serious ROB) there is only one study of adequate quality and -2 for imprecision sample size <50
Measurement error	Valentin and Maribo (2014) [68] (Ext)	Adequate	MIC (?)	**Indeterminate (?)**	**Not graded**
**Digital loading cells- Asymptomatic individuals**					
(Reliability Intra-rater).	Andre et al (2012) [36] (Rot.)	Inadequate	+		
	Essendrop, Schibye, and Hansen (2001) [40] (Ext, Flex)	Inadequate	+	**Sufficient (+)**	**High quality evidence** **(**There are multiple studies of at least adequate quality)
	Glenn et al. (2015) [41] (Flex)	Inadequate	+		
	Loss et al (2020) [48] (Flex)	Adequate	+		
	Mesquita et al (2019) [49] (Ext, Flex)	Inadequate	+		
	Paalanne et al (2009) [51] (Ext, Flex, R & L, Rot)	Inadequate	+		
	Pitcher, Behm, and MacKinnon (2008) [54] (Ext)	Adequate	+		
	Steeves et al (2019) [62] (Flex, Rotational push, Rotational pull, Kayak-Stroke Simulation)	Adequate	+		
Reliability (Inter rater)	Paalanne et al (2009) [51] (Ext, Flex, R & L Rot)	Inadequate	+	**Sufficient (+)**	**Very low-quality evidence** (-3 Extremely serious ROB, as there is only one study of inadequate quality available, -2 for imprecision sample size <50)
Measurement error	Essendrop, Schibye, and Hansen (2001) [40] (Ext, Flex)	Adequate	MIC (?)	**Indeterminate (?)**	**Not graded**
	Mesquita et al (2019) [49] (Ext, Flex)	Inadequate	MIC (?)		
	Paalanne et al (2009) [51] (Ext, Flex, R & L Rot)	Inadequate	MIC (?)		
Construct validity (convergent validity)	Glenn et al. (2015) [41] (Flex) **Vs. 1 RM.**	Doubtful	+	**Sufficient (+)**	**Very low-quality evidence** (-2 Very serious ROB, there is only one study of doubtful quality available, and -2 for imprecision sample size <50)
	Loss et al (2020) [48] (Flex) **Vs. calibrated barbells**	Inadequate	+	**Sufficient (+)**	**Very low-quality evidence** (-3 extremely serious ROB, there is one study of inadequate quality, and -2 for imprecision sample size <50)
	Steeves et al (2019) [62] **Vs. 200-m race time**	Inadequate	?	**Indeterminate (?)**	**Not graded**
**Digital loading cells- Spinal musculoskeletal pain**					
*LBP* (Reliability (Intra rater).	Pitcher, Behm, and MacKinnon (2008) [54] (Ext)	Adequate	+	**Sufficient (+)**	**Very low-quality evidence** (-1 serious ROB as there is only one study of adequate quality, and -2 imprecision sample size <50)
**Specialized and commercialized equipment- Asymptomatic individuals**					
(Reliability (Intra-rater).	Demoulin et al (2006) [39] (Ext, Flex, Lat. Flex & Rot) **(David- back)**	Inadequate	ICC (?)	**Sufficient (+)**	**Very low-quality evidence** (-2 very serious ROB as there are multiple studies of inadequate quality, and -1 imprecision sample size <50–100)
	Kienbacher et al (2014) [46] (Ext, Flex, & Rot) **(David- back)**	Inadequate	+		
	Graves et al (1990) [42] **(MedX)**	Inadequate	ICC (?)	**Indeterminate (?)**	**Not graded**
	Roussel et al (2006) [55] **(Tergumed)**	Inadequate	ICC (?)	**Indeterminate (?)**	**Not graded**
	Udermann, Mayer, and Murray (2004) [59] (Ext) **(BackUp)**	Inadequate	+	**Sufficient (+)**	**Very low-quality evidence** (-3 Extremely serious ROB as there is only one study of inadequate quality available and -1 for imprecision sample size <50–100)
	Scheuer and Friedrich (2010) [56] **(Back- check)**	Doubtful	+	**Sufficient (+)**	**Very low-quality evidence** (-2 Very serious ROB, as there is only one study of doubtful quality available, and -2 imprecision sample size <50).
(Reliability Inter- rater).	Roussel et al (2006) [55] **(Tergumed)**	Inadequate	+	**Sufficient (+)**	**Very low-quality evidence** (Extremely serious ROB as there is only one study of inadequate quality available and imprecision sample size <50–100)
	Scheuer and Friedrich (2010) [56] **(Back- check)**	Inadequate	?	**Indeterminate (?)**	**Not graded**
	Demoulin et al (2006) [39] **(David- back)**	Inadequate	?	**Indeterminate (?)**	**Not graded**
Measurement error	Graves et al (1990) [42] **(MedX)**	Inadequate	(MIC?)	**Indeterminate (?)**	**Not graded**
	Kienbacher et al (2014) [46] **(David- back)**	Inadequate	(MIC?)	**Indeterminate (?)**	**Not graded**
Construct validity (convergent validity)	Conway et al (2016) [64] **Medx Vs. TEX.**	Inadequate	?	**Indeterminate (?)**	**Not graded**
**Specialized and commercialized equipment- Spinal musculoskeletal pain**					
CLBP (Reliability (Intra rater).	Kienbacher et al (2016) [65] **(David- back)**	Inadequate	+	**Sufficient (+)**	**Very low-quality evidence.** (-3 Extremely serious ROB as there is only one study of inadequate quality available)
(Reliability Inter- rater).	Roussel et al (2008) [67] **(Tergumed)**	Inadequate	+	**Sufficient (+)**	**Very low-quality evidence.** (-3 Extremely serious ROB as there is only one study of inadequate quality available and -2 for imprecision sample size <50).
Measurement error	Kienbacher et al (2016) [65] **(David- back)**	Inadequate	(MIC?)	**Indeterminate (?)**	**Not graded**
	Roussel et al (2008) [67] **(Tergumed)**	Inadequate	(MIC?)	**Indeterminate (?)**	**Not graded**
Construct validity (convergent validity)	Conway et al (2016) [64] **MedX Vs. TEX.**	Inadequate	-	**Indeterminate (?)**	**Not graded**
NP (Reliability (Intra rater).	Scheuer and Friedrich (2010) [56] **(Back- check)**	Doubtful	+	**Sufficient (+)**	**Very low-quality evidence.** (-2 Very serious ROB, as there is only one study of doubtful quality available, and -1 for imprecision sample size <50–100).
(Reliability (Inter- rater).	Scheuer and Friedrich (2010) [56] **(Back- check)**	Inadequate	ICC?	**Indeterminate (?)**	**Not graded**
**Field tests- Asymptomatic individuals**					
(Reliability intra rater)	Sell et al (2015) [57] **Medicine ball toss tests (MBTs).**	Adequate	+	**Sufficient (+)**	**Very low-quality evidence.** (-1 Serious ROB as there is only one study of adequate quality and -2 for imprecision sample size <50).
	Cowley et al (2009) [38] **Front abdominal power test [FAPT])**	Doubtful	+	**Sufficient (+)**	**Very low-quality evidence.** (-2 Very serious ROB as here is only one study of doubtful quality available and -2 for imprecision sample size <50).
	Ladeira et al (2005) [47] **Double leg lowering manuver (DLLM)**	Inadequate	+	**Sufficient (+)**	**Very low-quality evidence.** (-3 Extremely serious ROB as there is only one study of inadequate quality available and -2 for imprecision sample size <50).
Measurement Error	Cowley et al (2009) [38]	Doubtful	MIC?	**Indeterminate (?)**	**Not graded**
	Sell et al (2015) [57]	Adequate	MIC?	**Indeterminate (?)**	**Not graded**
Construct validity (convergent validity)	Ladeira et al (2005) [47] **Double leg lowering manuver (DLLM) Vs. HHD**	Very Good	-	**Insufficient (-)**	**Low quality evidence**. (-2 for Imprecision sample size <50).
Criterion validity	Sell et al (2015) [57] **Medicine ball toss tests (MBTs)** **Vs. (The Biodex System 3)**	Very Good	-	**Insufficient (-)**	**Low quality evidence**. (-2 Imprecision sample size <50).
**Specially developed tools- Asymptomatic individuals**					
Reliability (intra-rater)	Azghani et al (2009) [37] **Triaxial isometric trunk strength measurement system**	Inadequate	+	**Sufficient (+)**	**Very low-quality evidence.** (-3 Extremely serious ROB as there is only one study of inadequate quality available and -2 for imprecision sample size <50).
	Pienaar and Barnard (2017) [53] (Ext) **A pressure air biofeedback**	Inadequate	+	**Sufficient (+)**	**Very low-quality evidence** (-3 Extremely serious ROB as there is only one study of inadequate quality available and -2 for imprecision sample size <50).
	Kato et al (2020) [45] **Innovative exercise device**	Inadequate	+	**Sufficient (+)**	**Very low-quality evidence** (-3 Extremely serious ROB as there is only one study of inadequate quality available and -2 for imprecision sample size <50).
Reliability (inter-rater)	Kato et al (2020) [45]	Inadequate	+	**Sufficient (+)**	**Very low-quality evidence** (-3 Extremely serious ROB as there is only one study of inadequate quality available and -2 for imprecision sample size <50).
Measurement error	Azghani et al (2009) [37]	Inadequate	?	**Indeterminate (?)**	**Not graded**
	Pienaar and Barnard (2017) [53] (Ext)	Inadequate	?	**Indeterminate (?)**	**Not graded**
	Kato et al (2020) [45]	Inadequate	?	**Indeterminate (?)**	**Not graded**
Construct validity (convergent validity)	Pienaar and Barnard (2017) [53] (Ext) **A pressure air biofeedback Vs. EMG**	Doubtful	+	**Indeterminate (?)**	**Not graded**
Construct validity (discriminative validity)	Pienaar and Barnard (2017) [53] (Ext)	Inadequate	+	**Indeterminate (?)**	**Not graded**
Criterion validity	Sasaki et al (2018) [69] (Flex, Ext) **(PTMI). Vs. Kin Com Isokinetic dynamometer).**	Inadequate	+	**Sufficient (+)**	**Very low-quality evidence** (-3 Extremely serious ROB as there is only one study of inadequate quality available and -2 for imprecision sample size <50).
**Specially developed tools- Spinal musculoskeletal pain (LBP)**					
Reliability (intra-rater)	Pienaar and Barnard (2017) [53] (Ext)	Inadequate	+	**Sufficient (+)**	**Very low-quality evidence** (-3 Extremely serious ROB as there is only one study of inadequate quality available and -2 for imprecision sample size <50).
Measurement error	Pienaar and Barnard (2017) [53] (Ext)	Inadequate	MIC?	**Indeterminate (?)**	**Not graded**
Construct validity	Pienaar and Barnard (2017) [53] (Ext)	Doubtful	-	**Indeterminate (?)**	**Not graded**

Abbreviations: Ext = Extension, Flex = Flexion, Lat. Flex = lateral flexion, Rot = Rotation, R = Right, L. = Left, HHD = Hand-held dynamometer, BC = Back-Check, + = Sufficient, = Indeterminate, MIC = Minimal important change, 1 RM. = one repetition maximum, m = meter, TEX = Thoracic extension endurance test, EMG = electromyography, PTMI = Portable Trunk muscle torque Measurement Instrument, LBP = low back pain.

### Synthesis of results

Results of the overall evidence of measurement properties against the COSMIN updated criteria and GRADE approach are presented in **Table 3**. In line with the COSMIN recommendation and the lack of information regarding the MIC for absolute reliability, the measurement error was not graded.

#### Hand-held dynamometer (HHD)

*Asymptomatic individuals*. Eight studies evaluated the intra-rater reliability of HHD for trunk flexion, extension and side bending where the dynamometer was externally fixed [43, 44, 47, 52, 60] or held by the examiner [58, 61, 63]. All studies rated as sufficient for the COSMIN criteria for good measurement properties where the ICC ranged from 0.8 to1.00 except for flexion from supine position where ICC = 0.67 [63] and side bending with the HHD positioned at axilla level where the ICC ranged 0.53 to 0.77 [61]. Overall, very low-quality evidence, due to very serious ROB, indicated very little confidence in the intra-rater reliability estimates for the use of HHD.

The inter-rater reliability of HHD was measured in three studies [50, 61, 63], all with inadequate ROB and overall, very low-quality evidence indicates very little confidence in the inter-rater reliability estimate.

Six studies investigated criterion validity of the HHD compared to ID [43, 52, 58, 60, 63] with moderate quality evidence overall indicating moderate confidence in the criterion validity estimates of the HHD in measuring trunk flexion and extension strength. Only one study with extremely serious ROB revealed sufficient convergent validity of the HHD compared to Back-Check (BC) [44] with overall, very low quality evidence.

*Individuals with spinal pain*. One study measured intra-rater reliability of HHD in individuals with LBP [66], the study was rated as inadequate ROB, with a rating of sufficient reliability for flexion (ICC = 0.74) and insufficient for extension (ICC = 0.65). Overall, there was very low-quality evidence indicating very little confidence in the reliability of HHD in measuring trunk strength in individuals with LBP. One study measured the intra-rater reliability for the HHD in individuals with osteoporosis [68], with overall, very low quality evidence indicated very little confidence in the reliability estimates for the HHD within the osteoporotic population due to imprecision (sample < 50).

#### Digital loading cell

*Asymptomatic individuals*. Eight studies assessed the intra-rater reliability of digital loading cells [36, 40, 41, 48, 49, 51, 54, 62]. All reported sufficient intra-rater reliability for flexion, extension, and rotation against the COSMIN for good measurement properties where ICCs across all studies ranged from 0.75–1.00. Overall, high quality evidence indicates high confidence in the intra-rater reliability estimate for the loading cells in asymptomatic populations. Sufficient inter-rater reliability of the strain gauge dynamometer (ICC > 0.80) was reported in one study, with overall, very low quality evidence (extremely serious ROB and small sample size (n = <50) [51]. Three studies evaluated the measurement error using the LOA and SEM, two with inadequate ROB [49, 51] and one rated as adequate ROB [40]. As recommended by COSMIN, no overall grading was given due to lack of information regarding the MIC.

Regarding the construct validity, values obtained from the loading cell were correlated with those from the barbell’s weight, indicating sufficient validity for trunk flexion strength [48]. However, there was very low-quality evidence due to extremely serious ROB and imprecision. There was sufficient convergent validity (r = 0.70) between the abdominal test and evaluation systems tool (ABTEST) and one-repetition maximum (1RM) in measuring abdominal strength with overall, very low-quality evidence indicating little confidence in validity estimates of the the digital loading cells [41].

*Individuals with spinal pain*. Sufficient intra-rater reliability of extension strength was observed in individuals with LBP with the ICC = 0.80. However, this study was graded as very low-quality evidence due to serious ROB and imprecision [54].

#### Specialised and commercialised equipment

Many devices are available to train and measure trunk muscle strength, and a significant correlation was observed between David Back system^®,^ Tergumed^®^ and Schnell^®^ were r = 0.8 [70]. However, designs, stabilisation systems, and individual positioning varied from one device to another, leading to substantial inter-system comparisons [17]. Therefore, each PBOM was rated and graded separately in this review. Five machines were identified: David Back system^®^ (David Health Solutions Ltd, Helsinki), MedX^TM^ (Ocala, FL), Tergumed^®^, BackUp^TM^ lumbar extension dynamometer (Priority One Equipment, Grand Junction, CO) and Back Check 607.

*Asymptomatic individuals*. The intra-rater reliability of the David Back and BackUp devices were evaluated in three studies, all with inadequate ROB [39, 46, 59] and very low-quality evidence overall indicating very little confidence in their reliability estimates of trunk strength. A single study, with doubtful ROB, used the Back-Check device [56]; based on COSMIN criteria, good intra-rater reliability was reported (ICC>0.8) with overall very low quality evidence indicated by very serious ROB and imprecision.

Sufficient inter-rater reliability of the Tergumed^®^ dynamometer was indicated by ICCs ranging from 0.95–0.97 [55] with overall, very low-quality evidence. One study of inadequate quality evaluated the construct validity of the MedX dynamometer compared to a thoracic extension endurance test (TEX) in healthy individuals [64]. This study was rated indeterminate for the COSMIN criteria for good measurement as there was no hypothesis identified, and therefore this study was not graded.

*Individuals with spinal pain*. Sufficient intra-rater and inter-rater reliability of the David Back system [65] and Tergumed dynamometer [67] respectively to assess trunk flexion, extension, and rotation strength among individuals with LBP. Overall, very low-quality evidence overall indicated very limited confidence in the reliability estimates of both devices. Sufficient inter-rater reliability of the Back-check, which was used to measure the flexion, extension and side bending strength from a standing position in 53 individuals with neck pain. However, there was very low-quality evidence (as only one study of doubtful quality and small sample available) [56]. One study of inadequate quality evaluated the construct validity of the MedX dynamometer compared to a thoracic extension endurance test in individuals with LBP [64]. This study was rated indeterminate for the COSMIN criteria for good measurement properties as there was no hypothesis identified, and therefore this study was not graded.

#### Functional tests (field tests)

Medicine ball toss tests were used to assess flexion, extension, and rotation strength in 20 individuals and showed good intra-rater reliability (ICC˃0.80) [57]. The overall level of evidence was very low as there was only one study of adequate quality and imprecision. The medicine ball toss tests were compared to the Biodex dynamometer where the Pearson correlation coefficient ranged from -0.04 to 0.1 (insufficient correlation) and with low quality evidence overall. The front abdominal power test (FAPT) and double leg lowering manoeuvre (DLLM) both showed excellent intra-rater reliability against COSMIN criteria. However, very low-quality evidence exists overall due to the study’s ROB and imprecision [38, 47]. Insufficient validity (negative correlation r = -0.338 to -0.446) between DLLM and HHD with overall low-quality evidence [47].

#### Novel devices

A triaxial isometric trunk muscle strength measurement system measured all trunk movements from an upright standing position [37]. Sufficient reliability were reported with overall very low-quality evidence was observed due to extremely serious ROB and imprecision [37].

The pressure air biofeedback (PAB^®^) is an isometric muscle testing instrument with an air-filled elastic ball held between the participant’s thighs. The PAB device demonstrated excellent to almost perfect reliability for extension strength (ICC = 0.99) in asymptomatic individuals (n = 24) and individuals with LBP (n = 18). However, very low-quality indicating very little confidence of the reliability estimates [53]. The Portable Trunk muscle torque Measurement Instrument (PTMI) was sufficiently correlated with the KinCom ID for flexion (r = 0.8) and extension (r = 0.7) however, the overall quality was rated very low indicating very little confidence in the results [69]. The last device identified is similar to a sphygmomanometer, where an inflatable cuff is placed around the abdomen and a mechanical manometer used to measure the deference in pressure (force) between baseline and participant maximum abdominal contraction [45]. Sufficient reliability, ICC = 0.95 and ICC = 0.99 for intra-rater and inter-rater reliability respectively, was observed with overall very low quality evidence due to extremely serious ROB and imprecision [45].

## Discussion

This rigorous systematic review is the first to summarise practicable PBOM of trunk muscle strength and their measurement properties. Thirty-four studies and 15 PBOM were identified, categorised, and reported for their measurement properties.

### Spinal pain population

Few studies, 8 out of 32, investigated reliability in spinal pain populations, and just two [48, 59] investigated validity. This raises concern regarding any reported evaluation of efficacy of rehabilitation programs in individuals with spinal pain [60]. Assessing trunk strength typically requires maximum effort of the individual during the testing [71]. Fear of pain, pain on exertion, lack of motivation and other confounding factors may affect the validity of the trunk strength testing in individuals with LBP. Pain is considered by some as a contraindication to maximum muscle strength testing [71] and may partly explain the paucity of research in spinal pain populations. Another possible reason is that in research settings, the ID is widely used to measure the trunk strength capacity in people with LBP [7, 72, 73]. As the current evidence highlighted the link between decreased trunk muscle strength and LBP [9, 74], review findings support the need for more high quality studies exploring the psychometric properties of practicable PBOM of trunk strength in spinal pain populations.

### Performance‐based outcome measures PBOM

Digital loading cells can be considered a practicable and easy to use tool to evaluate flexion, extension, and trunk rotation strength in an asymptomatic population. However, in the absence of research in spinal pain population nor data on responsiveness, caution should be taken in drawing any conclusions with respect to their use in spinal pain populations. Additionally, very low quality evidence on the inter-rater reliability questions the confidence of findings where more than one examiner is involved.

Moderate quality evidence supports criterion validity of the HHD, in relation to the ID, for measuring trunk flexion and extension muscle strength in an asymptomatic population. This aligns with other research examining HHD for proximal and distal muscle strength assessment in all extremities [27]. Findings suggest the use of the HHD as a practicable alternative to an isokinetic device in asymptomatic individuals. However, caution must be taken before interpreting the results due to the absence of high quality studies on the reliability of the HHD and the responsiveness of the tool among a spinal pain population. The applicability of the HHD in practice was previously questioned due to the variability of the results with repeated measurements and the influence of the examiner strength, especially when measuring large and strong muscle groups [26]. This finding was also highlighted in this review were the inter-rater reliability of the HHD was inconsistent. Using the HHD fixed by the examiner to measure flexion and extension strength in healthy individuals exhibited inconsistent inter-rater reliability, which could be attributed to examiner strength variability [75]. Given that the examiner strength can threaten the reliability of the HHD, several studies in this review used external fixation techniques, which facilitate participant force generation and could be usefully recommended when using the HHD to assess the trunk muscle strength, especially if the examination is carried out by different raters. The included field tests show promising levels of intra-rater reliability, using easy to administer measures that required little or no equipment to enhance the strength evaluation in clinical or sports settings [76]. However, caution must be taken as both criterion and construct validity of the included field tests is lacking. Consequently, more high-quality studies to explore the validity of the field tests are needed. Some novel devices reviewed in this paper, which showed sufficient psychometric properties, are quite complex and custom-built, and are therefore poorly reproducible or not feasible in clinical environments. Additionally, it may be time-consuming especially for untrained individuals.

### Trunk movements

Most measures evaluated movements in the sagittal plane with fewer evaluating trunk side bending and rotation strength [36, 37, 39, 44, 46, 51, 55–57, 61, 62, 65, 67] (all with very low-quality evidence). This is in line with earlier research investigating trunk muscle strength [7, 77]. The abundance of research examining the trunk strength in the sagittal plane is unsurprising given the known correlation between the lumbar extensor musculature deconditioning and the development of LBP [8]. Trunk rotation however is essential for activities of daily activity and sporting tasks [78] but has been relatively under investigated [77]. Further inquiry may be useful given findings from epidemiological studies concluding that trunk rotation contributed to 11.4% of traumatic back injuries and 49% of non-traumatic back pain [79, 80].

### Testing protocols

This review found a large variety in the use of PBOM rendering comparison between studies challenging. Where different testing positions were used, different levels of intra-tester reliability were observed. Trunk flexion strength, measured by a HHD, at 30˚ flexion showed excellent reliability ICC _(2,1)_ = 0.9 compared to good reliability ICC _(2,1)_ = 0.67 in a neutral position [63]. The 30˚ flexion position enhances the MVC output as previously suggested [81]. Notwithstanding position, the location of applied resistance also yielded different reliability estimates. Two studies [41, 48] investigated trunk flexion strength, using the digital loading cells from the same position (supine with flexed hips and knees) but differed with regard to the line of resistance, one being level with axilla ICC _(2,1)_ = 0.99 and the other at the xiphoid process ICC_(2,1)_ = 0.75. This was also noted when measuring the side bending strength using a HHD, from a sitting position; higher intra-rater reliability was observed when the resistance applied at the mid trunk ICC = 0.80–0.88 compared to the level of axilla ICC = 0.5–0.7 [61]. The influence of different testing protocols on reported psychometric properties was also seen for isokinetic testing [24, 73]. The variability in testing protocols utilised and the overall level of evidence prevents clinically important conclusions from being made [82].

### Quality of the included studies

This review highlighted the number of methodological flaws in the included studies which therefore resulted in the rating of doubtful or inadequate risk of bias, with the overall quality for each measure being low or very low. For reliability studies, there was inappropriate description of the study design where there was a lack of explicit reporting of the stability of the participants and testing environment between sessions. Different protocols have been used in terms of the time interval between tests and re-test or between testers and this varied remarkably, from immediately consecutive measurements to >2 weeks. As previously recommended, the interval time between trials and between testing sessions should be long enough to avoid fatigue and short enough to not cause change in the construct being measured [34]. Therefore, studies were rated as inadequate on the ROB checklist, if the time was less than 2 minutes for the between trial interval and less than 15 min and more than 2 weeks for between sessions rest interval [33, 83, 84]. Reporting the expertise or training level of the examiners prior to the actual test was unclear, except in ten studies [39, 40, 46, 50, 60, 65, 66–69] and just nine studies considered and detailed testing order and randomisation [38, 40, 41, 57, 60, 61, 63, 66, 69]. To establish validity, it is necessary to include patients with spinal pain who are likely to undergo the same measurement in daily practice [85]. However, only eight studies included people with spinal pain [53, 54, 56, 64–68]. Sample size was another factor which contributed to downgrading due to imprecision (n< 50–100). Another methodological flaw, which is an important aspect of internal validity, is the blinding of the examiners for the results and/or status of the participants. Blinding was not well documented and only reported in five studies [39, 43, 55, 66, 67]. Statistical measures used to evaluate the psychometric properties are an important aspect of the ROB assessment. In keeping with COSMIN recommendations for reliability analysis, intraclass correlation coefficients, ideally ICC _(2,1)_, for continuous data and Kappa coefficient statistics for dichotomous/nominal and ordinal data was the standard [33]. This was not always followed in the included studies, and subsequently the overall conclusion was downgraded, even if the outcome measures exhibit sufficient reliability.

### Implications for clinical practice and research

The assessment of trunk strength enables coaches and clinicians to determine whether changes in muscle strength reflects a true gain or loss, or is a product of measurement error. Even though findings revealed high-quality evidence for intra-rater reliability of the digital loading cells and moderate-quality evidence for the HHD, the findings should be interpreted with caution given a paucity of evidence derived from people with spinal pain.

Further high-quality studies using appropriate study designs and detailed testing protocols to standardise testing are needed to advance our understanding of practicable PBOM of trunk muscle strength, especially among a spinal pain population taking into account different levels of strength. The lack of studies measuring the responsiveness is a concern when considering the use of measures in spinal pain individuals undergoing rehabilitation and warrants immediate investigation. The review findings further highlighted the need to test the psychometric properties of measures evaluating trunk rotation and side bending strength; the majority of studies investigated sagittal plane motion.

### Strengths and limitations

This review was conducted according to a registered and published protocol and followed the COSMIN methodology and recommendations. Bias was minimised, where two reviewers independently conducted all stages of this review. Despite this, some limitations that need to be acknowledged. The current review included studies that evaluated the trunk muscle strength in adults only >18 years old, which prevents the generalisability of the findings to younger populations. The rating approach to assess the methodological quality of the included studies was based on the lowest score principle, this may underestimate the overall quality of studies and subsequently downgrade the overall quality of evidence. This approach was strictly in line with the COSMIN recommendations to obtain a high standard for methodological design and reporting of psychometric properties studies.

## Conclusion

The digital loading cells and the Hand-held dynamometer are objective and easy to use tools in everyday clinical practice. However, further studies are needed to investigate their psychometric properties in individuals with spinal pain to provide the practitioner with the most optimal tool to use with confidence. Review findings highlight gaps in the current evidence base of trunk strength measurement, notably a paucity of studies in pain populations and an absence of investigation of responsiveness; both of which are required to inform precision in clinical practice. Given the overall level of evidence, and the heterogeneity of methods and protocols used to measure trunk muscle strength, no recommendations regarding the optimal practicable outcome measure of trunk muscle strength can be made.

## Supporting information

S1 Table Preferred reporting items for systematic reviews and meta-analyses checklist-PRISMA. (PDF)

S2 Table COSMIN definitions of measurement properties. (PDF)

S1 Fig MEDLINE full search string. (PDF)

S1 Appendix Risk of bias assessment of both reviewers-reliability. (XLSX)

S2 Appendix Risk of bias assessment of both reviewers-measurement error. (XLSX)

S3 Appendix Risk of bias assessment of both reviewers-validity. (XLSX)

S4 Appendix Criteria for good measurement properties and overall quality of evidence (GRADE) of both reviewers. (XLSX)
