# Supplementary material for: Practicable performance-based outcome measures of trunk muscle strength and their measurement properties: A systematic review and narrative synthesis
Source: PLoS One. 2022 Jun 17;17(6):e0270101. doi: 10.1371/journal.pone.0270101 (PMC9205516; doi:10.1371/journal.pone.0270101)
Supplement: S1 Fig — (PDF) [file pone.0270101.s003.pdf]

MEDLINE full search string.

Set	Search Statement	Annotations	Insert	Edit	Delete
1.	Trunk musc* strength.ti,ab.				
2.	Trunk musc* power.ti,ab.				
3.	Trunk musc* torque.ti,ab.				
4.	Torso strength.ti,ab.				
5.	Torso power.ti,ab.				
6.	Isometric trunk strength.ti,ab.				
7.	Isotonic trunk strength.ti,ab.				
8.	Static trunk strength.ti,ab.				
9.	Back musc* strength.ti,ab.				
10.	Back exten* strength.ti,ab.				
11.	Lumbar exten* strength.ti,ab.				
12.	Lumbar exten* torque.ti,ab.				
13.	Abdomin$ musc* strength.ti,ab.				
14.	Trunk Max* contract*.ti,ab.				
15.	Trunk MVC.ti,ab.				
16.	Trunk flex* strength.ti,ab.				
17.	Trunk flex* power.ti,ab.				
18.	Trunk flex* torque.ti,ab.				
19.	Trunk forward bend* strength.ti,ab.				
20.	Trunk forward bend* power.ti,ab.				
21.	Trunk forward bend* tourque.ti,ab.				
22.	Trunk extens$ strength.ti,ab.				
23.	Trunk extens$ power.ti,ab.				
24.	Trunk extens$ torque.ti,ab.				
25.	(Trunk rota* strength or Trunk rota* power or Trunk rota* torque).ti,ab.				
26.	(Trunk lateral$ flex* strength or Trunk lateral$ flex* power or Trunk lateral$ flex* torque).ti,ab.				
27.	(Trunk lateral$ bend* strength* or Trunk lateral$ bend* power or Trunk lateral$ bend* torque).ti,ab.				
28.	(core strength or core power or core torque).ti,ab.				
29.	1 or 2 or 3 or 4 or 5 or 6 or 7 or 8 or 9 or 10 or 11 or 12 or 13 or 14 or 15 or 16 or 17 or 18 or 19 or 20 or 21 or 22 or 23 or 24 or 25 or 26 or 27 or 28				
30.	(Spinal musculoskeletal pain or musculoskeletal disease or musculoskeletal dysfunction* or spin* MUSCU* pain).ti,ab.				
31.	(Low* back pain or LBP or Chronic Low* back pain or CLBP).ti,ab.				
32.	(lumbago or dorsalgia).ti,ab.				
33.	back disorder$.ti,ab.				
34.	(lumbar adj pain).ti,ab.				
35.	(slipped adj disc).ti,ab.				
36.	(slipped adj disk).ti,ab.				
37.	(prolap* adj disc).ti,ab.				
38.	(prolap* adj disk).ti,ab.				
39.	Spin*osteoarthrit$.ti,ab.				
40.	Spine osteoarthrit$.ti,ab.				
41.	spine spondylitis.ti,ab.				
42.	spine spondylosis.ti,ab.				
43.	(spine degenerative adj joint adj disease).ti,ab.				
44.	(Neck pain or Cervical pain or Chronic neck pain or CNP or cervicogenic).ti,ab.				
45.	(Thoracic spine pain or Mid back pain).ti,ab.				
46.	(Healthy adult* or Normal adult* or A symptomatic adult* or Physically active adult* or Athlete*).ti,ab.				
47.	30 or 31 or 32 or 33 or 34 or 35 or 36 or 37 or 38 or 39 or 40 or 41 or 42 or 43 or 44 or 45 or 46				
48.	(Performance-based tool* or performance-based test* or Clinical-based tool*).ti,ab.				
49.	(field- based test* or Assessment).ti,ab.				
50.	(Quantitative assessment or measurement* or evaluate$).ti,ab.				
51.	(instrument$ or tool* or test$).ti,ab.				
52.	(Manual* musc* test$ or MMT).ti,ab.				
53.	(mechanic* or Hand-held dynamometer* or HHD or Strain-gauge test*).ti,ab.				

54. 48 or 49 or 50 or 51 or 52 or 53
55. 29 and 47 and 54
56. (Validation Studies or Comparative Study).pt.
57. exp Psychometrics/
58. psychometr*.ti,ab.
59. (clinimetr* or clinometr*).tw.
60. outcome assessment.ti,ab. or outcome measure*.tw. or exp Observer Variation/ or observer variation.ti,ab.
61. exp Health Status Indicators/
62. exp Reproducibility of Results/
63. reproducib*.ti,ab.
64. exp Discriminant Analysis/
65. (reliab* or unreliab* or valid* or coefficient or homogeneity or homogeneous or internal consistency).ti,ab.
66. (cronbach* and (alpha or alphas)).ti,ab.
67. (item and (correlation* or selection* or reduction*)).ti,ab.
68. (agreement or precision or imprecision or precise values or test-retest).ti,ab.
69. (test and retest).ti,ab.
70. (reliab* and (test or retest)).ti,ab.
71. (stabil* or interrater or intrarater or intrarater or intertester or intratester or interobserver or interobserver or intraobserver or intertechnician or intratechnician or interexaminer or intraexaminer or interassay or intraassay or interindividual or intraindividual or interparticipant or intraparticipant or kappa or kappas or repeatab*).ti,ab.
72. ((replicab* or repeated) and (measure or measures or findings or result or results or test or tests)).ti,ab.
73. (generaliza* or generalisa* or concordance).ti,ab.
74. (intraclass and correlation*).ti,ab.
75. (item discriminant or interscale correlation* or error or errors).mp. or individual variability.ti,ab. [mp=title, abstract, original title, name of substance word, subject heading word, floating sub-heading word, keyword heading word, organism supplementary concept word, protocol supplementary concept word, rare disease supplementary concept word, unique identifier, synonyms]
76. variability.mp. and (analysis or values).ti,ab. [mp=title, abstract, original title, name of substance word, subject heading word, floating sub-heading word, keyword heading word, organism supplementary concept word, protocol supplementary concept word, rare disease supplementary concept word, unique identifier, synonyms]
77. (uncertainty and (measurement or measuring)).ti,ab.
78. (standard error of measurement or sensitiv* or responsive*).ti,ab.
79. ((minimal or minimally or clinical or clinically) and (important or significant or detectable) and (change or difference)).ti,ab.
80. (small* and (real or detectable) and (change or difference)).ti,ab.
81. 56 or 57 or 58 or 59 or 60 or 61 or 62 or 63 or 64 or 65 or 66 or 67 or 68 or 69 or 70 or 71 or 72 or 73 or 74 or 75 or 76 or 77 or 78 or 79 or 80
82. 55 and 81
83. limit 82 to english language
